# Factors related to the use of reperfusion strategies in elderly patients with acute myocardial infarction

**DOI:** 10.1186/1749-8090-9-111

**Published:** 2014-06-20

**Authors:** Gui-yan Yi, Xing-guang Zhang, Jian Zhang, Xian Wang

**Affiliations:** 1 Department of Cardiology, Beijing military general hospital, No. 5 Nan Men Cang Dongcheng Distrct, 100700 Beijing, China; 2 Department of Endocrine, Beijing military general hospital, 100700 Beijing, China

**Keywords:** Acute myocardial infarction, Elderly, Percutaneous coronary intervention, Thrombolysis, Influencing factors

## Abstract

**Background:**

About eighty percent of acute myocardial infarction (AMI) cases occur in the elderly, we aim to examine the use of reperfusion strategies in elderly patients (≥65 years) with AMI and to investigate the factors affecting the use of these strategies.

**Methods:**

A total of 352 consecutive elderly patients (≥65 years) with ST-elevated AMI (STAMI) were admitted, they were divided into 2 groups based on reperfusion treatment (thrombolysis or percutaneous coronary intervention, PCI): reperfusion therapy group (n = 268) and non-reperfusion therapy group (n = 84). Demographic and medical data were collected for comparison. Odds ratios (*OR*) and 95% confidence interval (*C.I*.) were calculated directly from the estimated regression coefficients.

**Results:**

About 76.1% of the elderly patients with AMI received reperfusion treatment (62.5% received PCI, and 13.6% received thrombolysis). Stepwise Logistic regression analysis revealed that a patient age ≥75 years (95% *CI*: 0.194 ~ 0.590, *OR* = 0.338, *P* = 0.000) and medical history of angina (95% *CI*: 0.281 ~ 0.928, *OR* = 0.501, *P* = 0.014) were determining factors for receiving less reperfusion therapy. Complications including right ventricular myocardial infarction (MI) (95% *CI*: 1.618 ~ 12.907, *OR* = 4.472, *P* = 0.003), unbearable symptoms (95% *CI*: 1.132 ~ 3.928, *OR* = 1.839, *P* = 0.021) and medical insurance (95% *CI*: 1.313 ~ 4.524, *OR* = 2.429, *P* = 0.004) were independent predictors of reperfusion therapy. The reperfusion therapy subset analysis revealed that intracranial hemorrhage (2.7% vs. 8.3%, *P* = 0.000), left ventricular ejection fraction (LVEF) <45% (13.2% vs. 29.2%, *P* = 0.019) and mortality rate within 1 year (2.7% vs. 6.3%, *P* = 0.045) were significantly decreased in the PCI group as compared with thrombolysis.

**Conclusion:**

Elderly patients with a medical history of angina, right ventricular MI, unbearable symptoms and medical insurance are likely be recipients of reperfusion strategies.

## Background

Acute myocardial infarction (AMI) or ‘heart attack’ is one of the top ten leading causes of death worldwide [1], about eighty percent of AMI cases occur in the elderly [2]. Therefore, a clinically relevant research for coronary heart disease in elderly patients is needed, especially for those with ST-elevated myocardial infarction (STEMI). STEMI, resulted from a rupture of coronary artery plaque followed by a thrombosis, could cause acute coronary artery blockages and flow interruption.

Effective and continued opening of the infarct related artery (IRA) is crucial to protecting left ventricular function and improving prognosis for patients with AMI [3,4]. Reperfusion therapy, including thrombolytic therapy and percutaneous coronary intervention (PCI), is effective in reducing the mortality rate of STEMI patients [5,6]. However, many elderly patients with AMI do not receive reperfusion therapy in a timely and effective manner. The purpose of this study was to survey the present situation of elderly patients with AMI in the Beijing area who accepted reperfusion therapy and to discuss the predictors of the reception of reperfusion therapy.

## Methods

### Patients

The study has been approved and registered by Beijing military general hospital in January 2005, the Ethics committee approved relating screening, treatment, and data collection of these patients, all subjects signed written informed consent form. All works were undertaken following the provisions of the Declaration of Helsinki.

The prospective survey-included patients consisted of 352 consecutive cases of elderly patients with STEMI (≥65 years) who were admitted to the coronary care unit of the Beijing military general hospital from February 2005 to February 2009.

These patients were divided into 2 groups based on their reception of reperfusion (thrombolysis or PCI) strategies: reperfusion therapy group (n = 268) and non-reperfusion therapy group (n = 84). The inclusion criteria were (1) the patient was ≥ 65 years of age; (2) the patient was admitted into the hospital within 24 h of MI symptoms that meet the diagnostic criteria of AMI [7,8], including continued chest pain over 30 min; an elevated ST-segment over 2 mm in at least two continuous precardial leads or over 1 mm in at least two continuous limb leads (II, III, aVF); and serum creatine kinase isoenzymes (CK-MB) levels double that of the normal or upper limit; and (3) the patient provided written informed consent for the study. The exclusion criteria were (1) AMI occurred after admission for other diseases; (2) the clinical state of the patient did not allow for inclusion, as decided by a physician; and (3) the patient did not agree to participate in the study.

### Data collection

Investigators in the study were given uniform training in filling out the survey form. The survey contents included (1) basic clinical characteristics including their age, gender, marital status, education level, income, health insurance, cardiovascular risk factor and cardiovascular health history; (2) onset background factors, including ① location: either at home or elsewhere; ② time: daytime was referred to as 6:01 ~ 19:59 and nighttime was referred to as 20:00 ~ 6:00; and ③ whether anyone else was present during the attack; (3) Symptoms; (4) time period from the onset of symptoms to admission; and (5) type of reperfusion therapy (thrombolysis or PCI).

### Statistical analysis

Data were all analyzed using SPSS 11.5statistics software. Quantifiable data between the 2 groups were compared using Chi-square tests; double tests were adopted in all tests of statistically significant differences, and *P* < 0.05 was used to determine statistical significance. The various related factors affecting reperfusion therapy in the patients were analyzed by a Logistic regression analysis of multiple factors (Forwald: Conditional method). Single factor analysis was performed on variables meaningful for regression analysis or significantly influencing reperfusion therapy, and odds ratios (*OR*) and 95% confidence intervals (*CI.*) were calculated from the estimation of regression coefficients.

## Results and discussion

### Results

#### Comparisons of social population data, risk factors and background between the 2 groups

The social population data, risk factors, medical history and attack background of 2 groups were collected in Table 1. As we can see, most of the patients in the non-reperfusion therapy group was elderly (77 ± 7 vs. 73 ± 5 years, *P* = 0.000) and had a history of angina (*OR* = 0.492, *P* = 0.006). Most of the patients in the reperfusion therapy group had medical insurance (*OR* = 2.393, *P* = 0.002), high incomes (*OR* = 1.521, *P* = 0.032) and a well-educated background (*OR* = 1.424, *P* = 0.049).

**Table 1:** Demographic characteristics of patients in the reperfusion therapy (RTG) group and non RTG group

**Variables**	**RTG group (n = 268), n (%)**	**Non RTG group (n = 84), n (%)**	***P*** **values**	***OR*** **values**	**95%** ***C.I.***
Social descriptive variables					
Ages (mean ± SD,years)	73 ± 5	77 ± 7	0.000		
Female	91(34.0%)	33(39.2%)	0.246	0.747	0.446-1.229
Married	221(82.5%)	59(70.2%)	0.004	2.383	1.332-4.268
Education level			0.049	1.424	1.007-1.946
Primary school	115(42.9%)	45(53.6%)			
Middle school	55( 20.5%)	15(17.9%)			
Undergraduate/graduate	98(36.8%)	24(28.6%)			
Salaries (RMB/ month)			0.032	1.521	1.038-2.358
<500	31(11.6%)	21(25.0%)			
500 ~ 2000	153(57.1%)	43(51.2%)			
2000 ~ 5000	70(26.1%)	16(19.0%)			
>5000	14(5.2%)	4(4.8%)			
Medical insurance	218(81.3%)	55(65.5%)	0.002	2.393	1.427-4.397
Being professionally trained	38(14.2%)	8(9.5%)	0.091	2.251	0.831-5.416
Cardiovascular risk factors					
Hypertension	160(59.7%)	58(69.0%)	0.226	0.636	0.419-1.092
Diabetes	63(23.5%)	25(29.8%)	0.398	0.740	0.432-1.299
Hyperlipemia	59(22.0%)	18(21.4%)	0.860	0.992	0.529-1.820
Family history of coronary artery disease	19(7.1%)	3(3.6%)	0.229	2.570	0.551-12.203
Smoking	101(37.7%)	20(23.8%)	0.062	1.812	0.992-3.021
Medical histories					
MI, myocardial infarction	28(10.4%)	11(13.1%)	0.098	0.623	0.276-1.214
Angina	83(31.0%)	34(40.5%)	0.006	0.492	0.299-0.874
CHF, Chronic heart failure	16(5.6%)	4(4.8%)	0.289	0.508	0.132-1.892
Stroke	41(15.3%)	15(17.9%)	0.231	0.696	0.368-1.321
Backgrounds of MI					
Attack at night	89(33.2%)	32(38.1%)	0.261	0.794	0.456-1.902
Attack at home	231(86.2%)	73(86.9%)	0.872	1.069	0.452-2.349
Persons present at onset	222(82.8%)	71(84.5%)	0.325	1.419	0.781-2.283
Emergency medical services	128(47.8%)	42(50.0%)	0.568	1.119	0.730-1.952
ECG outside of hospital	118(44.0%)	34(40.5%)	0.258	1.302	0.817-2.354

#### Comparisons of symptom types and clinical characteristics between the 2 groups

The symptom types and clinical characteristics of the two patient groups were collected in Table 2. Most of the patients in reperfusion therapy group have unbearable symptoms (*OR* = 2.272, *P* = 0.002), polypnea (*OR* = 0.530, *P* = 0.036) and excessive perspiration (*OR* = 1.891,*P* = 0.021).

**Table 2:** Comparisons of symptom types and clinical characteristics between RTG and non RTG patients

**Variables**	**RTG group (n = 268), n (%)**	**Non RTG group (n = 84), n (%)**	***P*** **values**	***OR*** **values**	**95%** ***C.I.***
**Symptom types**					
Chest pain	227 (84.7%)	68 (81.0%)	0.261	1.461	0.754-2.981
Vomiting	116 (43.3%)	27 (32.1%)	0.191	1.447	0.891-2.420
Fears	48 (17.9%)	12 (14.3%)	0.616	1.272	0.689-2.989
Unbearable symptoms	198 (73.9%)	46 (54.8%)	0.002	2.272	1.322-3.694
Polypnea	49 (18.3%)	22 (26.2%)	0.036	0.530	0.291-0.983
Excessive perspiration	198 (73.9%)	50 (59.5%)	0.021	1.891	1.121-3.219
**Clinical characteristics**					
Antetheca MI	139 (51.9%)	50 (59.5%)	0.367	1.364	0.869-2.292
Right ventricle MI	60 (22.4%)	4 (4.8%)	0.000	4.615	1.692-11.234
Klliip ≥2 levels	49 (18.3%)	15 (17.9%)	0.366	0.921	0.737-1.311
Years of age ≥75	132 (49.3%)	55 (65.5%)	0.000	0.318	0.297-0.641

### Multivariable analyses of the factors influencing reception of reperfusion therapy in elderly patients with AMI

Results showed that five factors, including age (*OR* = 0.338, *P* = 0.000), a history of angina (*OR* = 0.501, *P* = 0.014), right ventricle MI (*OR* = 4.472, *P* =0.003), unbearable symptoms (*OR* = 1.839, *P* =0.021), medical insurance (*OR* = 2.429, *P* =0.004) were statistically significance in predicating whether the patients of AMI received reperfusion therapy (Table 3).

**Table 3:** Multivariable analyses of factors affecting the reception of reperfusion therapy in elderly patients with AMI

**Variables**	**Β values**	***P*** **values**	***OR*** **values**	**95% *C.I *.**
Years of age ≥75	−1.252	0.000	0.338	0.194-0.590
History of angina	−0.697	0.014	0.501	0.281-0.928
Right ventricle MI	1.523	0.003	4.472	1.618-12.907
Unbearable symptoms	0.623	0.021	1.839	1.132-3.928
Medical insurance	0.882	0.004	2.429	1.313-4.524

### Reperfusion therapy subset analysis

The patients were divided into 2 subgroups based on the reception of reperfusion therapy methods: PCI (n = 220) and thrombolysis (n = 48) groups. Results showed the intracranial hemorrhage (2.7% vs 8.3%, *P* = 0.000), left ventricular ejection fraction (LVEF) < 45% (13.2% vs 29.2%, *P* = 0.019) and mortality rate within 1 year (2.7% vs 6.3%, *P* = 0.045) were significantly decreased in the PCI group as compared with the thrombolysis group (Table 4). Nonfatal MI (*P* = 0.292) and revascularization (*P* = 0.239) showed no statistically significant difference between the PCI and thrombolysis groups.

**Table 4:** Reperfusion therapy subset analyses in elderly patients with AMI

**Variables**	**PCI group (n = 220)**	**Thrombolysis group (n = 48)**	***P*** **values**
Ages (mean ± SD, yrs)	70.9 ± 4.32	69.7 ± 4.01	0.782
Males, n (%)	144 (65.5%)	33 (68.8%)	0.641
**Cardiovascular risk factors, n (%)**			
Hypertension	131 (59.5%)	29 (60.4%)	0.279
Diabetes	52 (23.6%)	11 (22.9%)	0.321
Hyperlipidemia	51 (23.2%)	8 (16.7%)	0.417
Smoking	86 (39.1%)	15 (31.2%)	0.312
**Medical histories, n (%)**			
MI	22 (10%)	6 (14%)	0.087
Chronic heart failure (CHF)	13 (5.9%)	3 (26%)	0.098
Stroke	31 (12.7%)	10 (20.8%)	0.059
**Clinic events, n (%)**			
Intracranial hemorrhage	6 (2.7%)	4 (8.3%)	0.000
Nonfatal MI	19 (8.6%)	5 (10.4%)	0.292
Revascularization	25 (11.4%)	6 (12.5%)	0.239
LVEF < 45%	29 (13.2%)	14 (29.2%)	0.019
Mortality within 1 year	6 (2.7%)	3 (6.3%)	0.045

CABG = coronary arteries bypass grafting.

## Discussion

Recently, the American College of Cardiology/American Heart Association (ACC/AHA) and European Society of Cardiology (ESC) guidelines revealed that thrombolysis was at least as effective as balloon dilation for patients admitted within 3 h of the onset of MI if the time between admission and balloon dilation was over 90 min [9-11]. Therefore, it was of extreme significance for patients with AMI to receive timely and effective reperfusion therapy, which includes either thrombolysis or PCI. However, the current status of the reception of reperfusion therapy in elderly patients with AMI in China is unsatisfactory.

Single factor analysis of social population material, risk factors, medical history, attack background factors, symptom type and other clinical characteristics revealed in this study show that most of the patients in the non-reperfusion therapy group were elderly (77 ± 7 vs 73 ± 5 years on an average, *P* < 0.001) and had a history of angina (*OR* = 0.591, *P* = 0.014). For patients over 80 years of age, thrombolytic therapy is indeed risky; but PCI, when performed by an experienced operator in a catheterization room, is one of the most effective measures for opening infarction associated vessels, and is a vital therapy for protecting the cardiac function and improving the survival rate of elderly patients with AMI. Some patients with a history of angina do not receive effective reperfusion therapy because (1) these patients were accustomed to the onset of chest pain can miss the golden window for PCI by dismissing their symptoms or (2) symptoms of angina masked those of AMI, which is especially common in patients with diabetes. Most of the patients in the reperfusion therapy group had medical insurance (*OR* = 2.429, *P* = 0.004), higher economic income, (*OR* = 1.521, *P* = 0.032) and a well-educated background (*OR* = 1.424, *P* = 0.049). Patients with medical insurance and higher income could receive timely and effective reperfusion therapy, suggesting whether the capacity for timely payment of medical expenses was also a factor affecting the reception of reperfusion therapy in elderly patients with AMI. This finding could potentially have an impact on medical insurance systems for the management of elderly patients with AMI. Well educated elderly patients with AMI could receive more effective reperfusion therapy because (1) they realized at once that AMI had occurred and that their lives were in danger; or (2) they were aware that PCI treatment of coronary heart disease had the ability to effectively open coronary artery infarctions. Therefore, popularization of coronary heart disease to improve public understanding of different types of reperfusion therapy, such as PCI, is of great significance.

Elderly patients with AMI who exhibited onset symptoms such as excessive perspiration (*OR* = 2.272, *P* = 0.002), dyspnea (*OR* = 0.530, *P* = 0.036) and unbearable symptoms (*OR* = 1.891, *P* = 0.021) were more likely to receive reperfusion therapy; This was likely related to a realization of the severity and life-threatening nature of their symptoms. The multi-factor analysis showed that five factors, consisting of age, a history of angina, right ventricular MI, unbearable symptoms and medical insurance, were independent predictors of the reception of reperfusion therapy. Patients with AMI, older than 75 years of age with a history of angina, were less likely to receive reperfusion therapy, while those with right ventricular MI, unbearable symptoms and medical insurance were more likely to receive therapy.

Additionally, this study did not show a statistically significant difference (*OR* = 1.119, *P* = 0.568) in alerting Emergency Medical Services (EMS) between the two groups; however, the importance of EMS should be emphasized in the treatment of elderly patients with AMI. EMS offer potential advantages toward reducing mortality: (1) EMS can implement life support in the case of cardiac arrest outside of the hospital. In the United Kingdom Heart Attack Study (UKHAS), among the 1829 cases of EMS-transported patients, 111 cases were resuscitated successfully by aid workers and 74% of the patients were diagnosed with ventricular tachycardia or fibrillation [12]. (2) EMS can transport patients to a hospital capable of reperfusion therapy rapidly; (3) EMS can perform electrocardiographic (ECG) examination and offer a diagnosis on the spot, which is significant for reducing pre-hospital delays; (4) EMS can inform doctors and nurses in the emergency room or catheterization room while in transit; and (5) a well-equipped EMS can carry out the thrombolytic therapy at the home of the patient or in the ambulance, and studies have shown that nosocomial mortality declines significantly for pre-hospital thrombolytic patients [13].

Previous clinical trials have shown that thrombolysis significantly increases fatal complications such as bleeding, especially intracranial hemorrhage, and should not be performed on elderly patients over 80 years of age with STEMI [14]. Facilitated PCI was used to carry out thrombolysis or combined anticoagulant therapy before PCI, and was suspended because of the increased mortality and bleeding complications in AMI patients. In recent years, many clinical trials have shown that direct PCI, especially with stents, was superior to thrombolytic therapy for improving left ventricular function, reducing the mortality rate within one year and reducing intracerebral hemorrhage for elderly patients with STEMI, as long as reperfusion is performed within the golden period window [15-17]. The fatality rate decreases significantly for elderly patients with STEMI over 75 years of age as a result of cardiogenic shock if direct PCI, aided by a percutaneous intra-aortic balloon pump (IABP), is performed [10].

The reperfusion therapy subset analysis in this study revealed that intracranial hemorrhage (2.7% vs. 8.3%, *P* = 0.000), LVEF < 45% (13.2% vs. 29.2%, *P* = 0.019) and mortality rate within one year (2.7% vs. 6.3%, *P* = 0.045) were significantly decreased in the PCI group, as compared with thrombolysis. This trend was identical to those reported in literature, suggesting that direct PCI is superior to thrombolytic therapy for protecting left ventricular function, reducing bleeding complications and improving one year survival rates in elderly patients with AMI.

## Conclusion

Direct PCI could become an important reperfusion method for the treatment of elderly patients with STAMI with continued improvements in equipment and increases in the number of experienced physicians. However, more studies with larger sample sizes are required to explain the results presented in this paper.

## Abbreviations

ACC/AHA: American College of Cardiology/American Heart Association; AMI: Acute myocardial infarction; *C.I*.: Confidence interval; CK-MB: Creatine kinase isoenzymes; ECG: Electrocardiography; EMS: Emergency medical services; ESC: European society of cardiology; IRA: Infarct related artery; LVEF: Left ventricular ejection fraction; MI: Myocardial infarction; STEMI: ST-elevated myocardial infarction; STAMI: ST-elevated acute myocardial infarction; PCI: Percutaneous coronary intervention; OR: Odds ratios; UKHAS: United Kingdom heart attack study.

## Competing interests

The authors declare that they have no competing interests.

## Authors’ contributions

WX and YGY defined the research theme and designed methods; YGY carried out the experiments; ZXG and ZHB analyzed the data, interpreted the results. ZLJ and RXX co-worked on associated data collection and their interpretation. All authors read and approved the final manuscript.
